# Association of Child Masking With COVID-19–Related Closures in US Childcare Programs

**DOI:** 10.1001/jamanetworkopen.2021.41227

**Published:** 2022-01-27

**Authors:** Thomas S. Murray, Amyn A. Malik, Mehr Shafiq, Aiden Lee, Clea Harris, Madeline Klotz, John Eric Humphries, Kavin M. Patel, David Wilkinson, Inci Yildirim, Jad A. Elharake, Rachel Diaz, Chin Reyes, Saad B. Omer, Walter S. Gilliam

**Affiliations:** 1 Yale School of Medicine, New Haven, Connecticut; 2 Department of Pediatrics, Yale School of Medicine, New Haven, Connecticut; 3 Yale Institute for Global Health, New Haven, Connecticut; 4 Department of Epidemiology, Mailman School of Public Health, Columbia University, New York, New York; 5 Tobin Center for Economic Policy, Yale University, New Haven, Connecticut; 6 Yale Child Study Center, Yale School of Medicine, New Haven, Connecticut; 7 Department of Economics, Yale University, New Haven, Connecticut; 8 Department of Epidemiology of Microbial Diseases, Yale School of Public Health, New Haven, Connecticut; 9 Yale School of Nursing, New Haven, Connecticut

## Abstract

**Importance:**

It is not known how effective child masking is in childcare settings in preventing the transmission of SARS-CoV-2. This question is critical to inform health policy and safe childcare practices.

**Objective:**

To assess the association between masking children 2 years and older and subsequent childcare closure because of COVID-19.

**Design, Setting, and Participants:**

A prospective, 1-year, longitudinal electronic survey study of 6654 childcare professionals at home- and center-based childcare programs in all 50 states was conducted at baseline (May 22 to June 8, 2020) and follow-up (May 26 to June 23, 2021). Using a generalized linear model (log-binomial model) with robust SEs, this study evaluated the association between childcare program closure because of a confirmed or suspected COVID-19 case in either children or staff during the study period and child masking in both early adoption (endorsed at baseline) and continued masking (endorsed at baseline and follow-up), while controlling for physical distancing, other risk mitigation strategies, and program and community characteristics.

**Exposures:**

Child masking in childcare programs as reported by childcare professionals at baseline and both baseline and follow-up.

**Main Outcomes and Measures:**

Childcare program closure because of a suspected or confirmed COVID-19 case in either children or staff as reported in the May 26 to June 23, 2021, end survey.

**Results:**

This survey study of 6654 childcare professionals (mean [SD] age, 46.9 [11.3] years; 750 [11.3%] were African American, 57 [0.9%] American Indian/Alaska Native, 158 [2.4%] Asian, 860 [12.9%] Hispanic, 135 [2.0%] multiracial [anyone who selected >1 race on the survey], 18 [0.3%] Native Hawaiian/Pacific Islander, and 5020 [75.4%] White) found that early adoption (baseline) of child masking was associated with a 13% lower risk of childcare program closure because of a COVID-19 case (adjusted relative risk, 0.87; 95% CI, 0.77-0.99), and continued masking for 1 year was associated with a 14% lower risk (adjusted relative risk, 0.86; 95% CI, 0.74-1.00).

**Conclusions and Relevance:**

This survey study of childcare professionals suggests that masking young children is associated with fewer childcare program closures, enabling in-person education. This finding has important public health policy implications for families that rely on childcare to sustain employment.

## Key Points

**Question:**

Is child masking associated with reduced COVID-19–related childcare program closures?

**Findings:**

In this survey study of 6654 childcare professionals from all 50 states, child masking at baseline (May 22 to June 8, 2020) was associated with a 13% reduction in program closure within the following year, and continued child masking throughout the 1-year study period was associated with a 14% reduction in program closure.

**Meaning:**

These results suggest that masking of children in childcare programs is associated with reduced program closures, supporting current masking recommendation in younger children provided by the Centers for Disease Control and Prevention.

## Teaser

This survey study assesses whether child masking policies are associated with COVID-19–related childcare program closures.

## Introduction

The COVID-19 pandemic and resulting childcare closures have left many parents and guardians struggling to find care for their children while continuing to work, leading to adverse mental health and financial outcomes for families.^1^ Thus, keeping childcare programs open safely is of paramount importance. Although exposure to childcare early in the pandemic demonstrated no increased risk of contracting COVID-19,^2^ the highly contagious B.1.617.2 (Delta) variant has increased community prevalence, and COVID-19 outbreaks in childcare and among younger children are now well described.^3,4,5^ Furthermore, the attack rate for the B.1.1.7 (Alpha) variant, another highly contagious strain, is similar for both children and adults during childcare outbreaks.^4^

Face masks reduce SARS-CoV-2 respiratory droplet transmission in the community and high-risk environments.^6,7^ In kindergarten through 12th grade schools, masks are part of successful risk mitigation bundles that facilitate a safe return to in-person education.^8,9,10,11^ Studies^8,9^ suggest that with strict masking policies social distancing can be safely reduced from 6 to 3 feet. However, child masking has not been studied in childcare, where children are typically younger than 5 years, social distancing is challenging, and adherence to masking is less than in older children.^12^ This gap in science is particularly problematic given current public debate regarding the benefits and risks of masking younger children not yet eligible for vaccination. We hypothesized that child masking, regardless of social distancing practices, is associated with reduced risk of a childcare program closing because of COVID-19 cases in either staff or children.

## Methods

We conducted a 1-year, prospective, longitudinal survey study of childcare professionals throughout the US and territories between May 22 to June 8, 2020 (baseline), and May 26 to June 23, 2021 (follow-up).^2,13^ Survey questions used for this analysis are found in the eAppendix in the Supplement. Data were deidentified before analysis, and the study was determined to be exempt by the institutional review board of the Yale School of Medicine. This study followed the American Association for Public Opinion Research (AAPOR) reporting guideline.

Baseline data were collected via Qualtrics survey from 19 114 participants actively providing childcare from May 22 to June 8, 2020. The data were identified through various childcare professional national databases and state childcare professional registries that consented to participate in a follow-up survey and collected all the required information at baseline, so that we could perform data analysis for this study. These national databases and state registries are described in detail in an earlier study.^2^ Of these childcare professionals, 16 630 consented to being contacted for a follow-up survey, with 7716 (46.4%) responding to the follow-up survey (Figure). Reasons for the lack of response included invalid email address (181 [1.1%]), duplicate emails (144 [0.9%]), and the email bouncing back because it was no longer on the system server (236 [1.4%]). The analysis sample was 6654 of responders who self-identified as childcare professionals, consented to participate, and provided follow-up data regarding COVID-19–related closures (Figure; Table 1).

**Figure.**
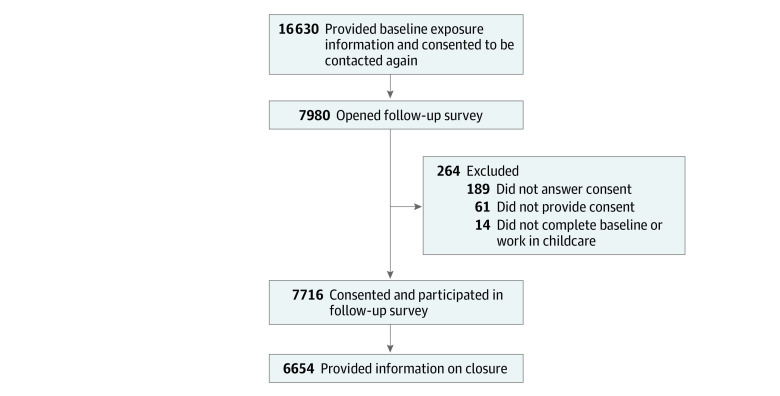
Number of Participants Eligible for and Included in the Analysis

**Table 1.** Characteristics of Childcare Programs at Baseline Comparing Responders and Nonresponders to the Follow-up Survey

Characteristic	No. (%)		χ^2^	*P* value	Cramér *V*^a^
	Responders (n = 7716)	Nonresponders (n = 8914)			
COVID-19 closure before baseline					
Yes	444 (5.8)	496 (5.6)	0.28	.59	.0041
No	7266 (94.2)	8410 (94.4)			
Masking at baseline					
Child					
Yes	772 (10.0)	1090 (12.2)	20.57	<.001	−.0352
No	6944 (90.0)	7823 (87.8)			
Adult					
Yes	2617 (33.9)	3159 (35.4)	4.25	.04	−.0160
No	5099 (66.1)	5754 (64.6)			
Childcare type					
Home based	3548 (46.0)	3820 (42.9)	16.34	<.001	.0314
Center based	4159 (54.0)	5082 (57.1)			
County COVID-19 cumulative death rate (deaths per 1000 population)					
Low (0-0.0561)	3033 (39.3)	3599 (40.4)	2.09	.35	.0112
Moderate (0.0564-0.2180)	2761 (35.8)	3147 (35.3)			
High (0.2184-13.5248)	1920 (24.9)	2163 (24.3)			
County median household income					
Low ($13 242-$54 976)	2504 (32.5)	3384 (38.0)	68.75	<.001	.0643
Medium ($54 979-$65 010)	2508 (32.5)	2856 (32.1)			
High ($65 027-$136 268)	2702 (35.0)	2669 (30.0)			

^a^ Cramér *V* is an effect size measure for χ^2^ tests of variable associations.

### Variables

The exposure variable was initially defined as all children (2 years and older) wearing a mask or facial covering at baseline (April 2020). We also assessed child masking during the past 15 days the program was open before survey completion at both baseline and follow-up 1 year later (see survey questions in the eAppendix in the Supplement). Covariates included other various infection mitigation strategies reported at baseline by childcare professionals to prevent transmission of COVID-19 (no = 0, yes = 1), including temperature and COVID-19 symptom screening, outside drop-off and pickup, and maintaining a distance of 6 ft between child seats and cots (6-ft distancing) (Table 2). These variables represent self-reported and observed practices as reported by the childcare professionals. The outcome was whether the childcare professional reported at follow-up that the program had ever experienced a COVID-19–related closure because of a child or staff case or suspected case of COVID-19 (COVID-19 closure; no = 0, yes = 1) in the interval between surveys (May 22 to June 8, 2020, and May 26 to June 23, 2021).

**Table 2.** Risk Mitigation Strategies Reported in Childcare Programs at Baseline and/or 1 Year Later in a Follow-up Survey

Mitigation strategies	No. (%) of respondents		
	Baseline (May 22-June 8, 2020)^a^	Follow-up (May 26-June 23, 2021)^b^	Both baseline and follow-up^c^
Screening (once per d)			
Children symptom screened	5291 (79.5)	5208 (82.6)	4476 (67.3)
Staff symptom screened	4988 (75.0)	4876 (77.3)	4061 (61.0)
Children’s temperatures	5021 (75.5)	4910 (77.9)	4111 (61.8)
Staff’s temperatures	4540 (68.2)	4445 (70.5)	3535 (53.1)
Masking			
Staff	2124 (31.9)	4063 (64.4)	1788 (26.9)
Child	572 (8.6)	2060 (32.7)	408 (6.1)
Social distancing			
6-ft Distancing	4338 (65.2)	3665 (58.1)	2913 (43.8)
Staggered arrival and departure	3174 (47.7)	2577 (40.9)	1830 (27.5)
Outdoor dropoff and pickup	3854 (57.9)	3666 (58.1)	2868 (43.1)

^a^ Transmission mitigation variables coded based on endorsement at baseline survey (May 22 to June 8, 2020; no = 0, yes = 1; n = 6654).

^b^ Transmission mitigation variables coded based on endorsement at follow-up survey (May 26 to June 23, 2021; no = 0, yes = 1; n = 6307).

^c^ Transmission mitigation variables coded based on endorsement at both baseline (May 22-June 8, 2020) and follow-up (May 26-June 23, 2021) (not endorsed at both = 0, endorsed at both = 1; n = 6654).

We calculated the COVID-19 prevalence between survey waves as the number of reported cases during the time window divided by the population of the county. Case data were extracted from the Johns Hopkins University dashboard,^14^ and the time window was defined as the time between the median dates the 2 surveys were open (May 27, 2020, and June 9, 2021). This variable was trichotomized into proportionally equal thirds to form a categorical variable representing low, medium, and high transmission rates.

### Statistical Analysis

We used a generalized linear model (log-binomial) with robust SEs to estimate risk ratios for the association among COVID-19 closure, child masking, and 6-ft distancing, controlling for other risk mitigation strategies and program and community characteristics presented in Table 2 and Table 3. We also tested in a separate model whether continued child masking and 6-ft distancing affected associations by coding the mitigation strategies in Table 2 as yes (1) if reported at both baseline and follow-up and no (0) if not. This method represents programs that reported practicing these activities at both time points, a proxy for continued practices with an assumption of no intermittent breaks, compared with programs that reported practicing mitigation strategies at any one time point or not at all. We also ran separate models with combined adult and child masking variable as the exposure variable coded as a categorical variable of 0 to 3, with 0 representing no masking, 1 representing adult masked but no child masking, 2 representing child masked but no adult masking, and 3 representing both masked. A 2-sided *P* < .05 was considered to be statistically significant.

**Table 3.** Outcome Measures and Childcare Program and Community Characteristics of the Follow-up Survey Respondents

Characteristic	No. (%) of respondents (N = 6654)
COVID-19 closure	
Did not close because of a COVID-19 case	3815 (57.3)
Closed because of a COVID-19 case	2839 (42.7)
Age, y	
18-24	136 (2.0)
25-34	786 (11.8)
35-44	1562 (23.5)
45-54	2107 (31.7)
55-64	1637 (24.6)
65-74	397 (6.0)
75-84	20 (0.3)
Race	
African American	750 (11.3)
American Indian/Alaska Native	57 (0.9)
Asian	158 (2.4)
Native Hawaiian/Pacific Islander	18 (0.3)
White	5020 (75.4)
Multiracial^a^	135 (2.0)
Prefer not to answer	516 (7.8)
Ethnicity	
Hispanic	860 (12.9)
Non-Hispanic	5659 (85.0)
Prefer not to answer	135 (2.0)
Local prevalence rates (cases per 100 000)^b^	
Low (<87.5)	2168 (32.9)
Moderate (87.5-109.6)	2198 (33.4)
High (>109.6)	2215 (33.7)
Childcare program type	
Home based or nanny	3068 (46.1)
Center based	3585 (53.9)
Center-based subtype^c^	
For profit	1959 (54.6)
Not for profit	799 (22.3)
School based	137 (3.8)
Head Start or Early Head Start	106 (3.0)
Drop-in	16 (0.4)
Faith based	451 (12.6)
Other or nonspecified	117 (3.3)
No. of children in program	
1-25	3355 (53.9)
26-50	711 (11.4)
51-100	1260 (20.3)
101-150	565 (9.1)
151-200	215 (3.5)
>200	113 (1.8)
No. of adults in program	
1-5	3417 (53.2)
6-10	685 (10.7)
11-20	1231 (19.2)
21-25	411 (6.4)
26-30	250 (3.9)
31-35	164 (2.6)
>35	262 (4.1)

^a^ Anyone who selected more than 1 race on the survey.

^b^ Cumulative COVID-19 cases between the median date of the 2 surveys in the program’s county per 100 000 population.

^c^ Of 3585 center-based programs.

## Results

This survey study included 6654 childcare professionals (mean [SD] age, 46.9 [11.3] years; 750 [11.3%] were African American, 57 [0.9%] American Indian/Alaska Native, 158 [2.4%] Asian, 860 [12.9%] Hispanic, 135 [2.0%] multiracial [anyone who selected >1 race on the survey], 18 [0.3%] Native Hawaiian/Pacific Islander, and 5020 [75.4%] White). Children older than 2 years accounted for 22 210 (66.4%) of the total childcare population served in these programs (specifically, 13 820 [33.7%] were 2 years and younger, 18 695 [45.6%] were 3-5 years of age, and 8515 [20.8%] were 6 years or older). The characteristics of the childcare programs and demographic characteristics of the respondents are shown in Table 1. Childcare programs of respondents did not differ significantly from nonrespondents on baseline county-level COVID-19 death rates or on whether the program ever closed because of COVID-19 before baseline. Respondents were more likely home based (paid childcare provided in a home) rather than center based (paid childcare provided in a childcare center) and in counties with higher mean annual household income and less likely to endorse masking at baseline, although the Cramér *V*, an effect size measure for χ^2^ tests of variable associations, was in the negligible range (<|0.10| for each).^15^ At follow-up, respondents reported that 2839 programs (42.7%) had closed because of COVID-19 (Table 3). Child masking increased from 572 programs (8.6%) at baseline to 2060 programs (32.7%) 1 year later, with 408 programs (6.1%) masking at both time points (Table 2). Changes in adherence to other practiced mitigation measures during the study period are described Table 2.

In multivariable analysis (Table 4), early adopting of child masking at baseline was associated with 13% lower risk of subsequent COVID-19 closure at follow-up (adjusted risk ratio [aRR], 0.87; 95% CI, 0.77-0.99; *P* = .04) compared with programs not practicing child masking. Conversely, 6-ft distancing was not associated with COVID-19 closures (aRR, 0.95; 95% CI, 0.89-1.02; *P* = .15). This finding translates into an absolute risk reduction of 5.8 percentage points (95% CI, 0.9-10.7 percentage points; *P* = .02) for programs that practiced child masking earlier in the pandemic.

**Table 4.** Association Between Reported Childcare Policies and Characteristics and Reported COVID-19–Related Childcare Closures

Characteristic	Baseline^a^		Baseline and follow-up^b^	
	aRR (95% CI)	*P* value	aRR (95% CI)	*P* value
Risk mitigation strategies				
Masking				
Child	0.87 (0.77-0.99)	.04^c^	0.86 (0.74-1.00)	.04^c^
Staff	0.97 (0.89-1.02)	.42	0.98 (0.91-1.06)	.64
6-ft Distancing	0.95 (0.89-1.02)	.15	0.93 (0.87-1.00)	.05^c^
Staggered arrival and departure	1.04 (0.98-1.11)	.21	1.04 (0.97-1.12)	.29
Outdoor drop-off and pickup	1.07 (1.00-1.14)	.04^c^	1.06 (1.00-1.14)	.07
Children symptom screened (once per d)	0.96 (0.85-1.09)	.54	1.02 (0.91-1.15)	.69
Staff symptom screened (once per d)	1.11 (0.98-1.25)	.11	1.07 (0.95-1.19)	.25
Children’s temperatures (once per d)	1.10 (0.97-1.24)	.12	1.05 (0.94-1.17)	.35
Staff’s temperatures (once per d)	0.95 (0.85-1.06)	.33	0.97 (0.87-1.07)	.53
Community and program variables				
Local prevalence rates (per 100 000 population)				
Low (<87.5)	1 [Reference]	NA	1 [Reference]	NA
Moderate (87.5-109.6)	1.08 (1.00-1.16)	.05	1.06 (0.98-1.14)	.14
High (>109.6)	1.16 (1.08-1.25)	<.001^c^	1.16 (1.08-1.25)	<.001^c^
Home based vs center based^d^	0.88 (0.74-1.04)	.14	0.92 (0.77-1.10)	.37
No. of children in program				
1-25	1 [Reference]	NA	1 [Reference]	NA
26-50	1.16 (0.97-1.39)	.11	1.15 (0.96-1.39)	.13
51-100	1.08 (0.88-1.31)	.43	1.07 (0.88-1.30)	.49
101-150	1.01 (0.82-1.25)	.93	1.01 (0.81-1.25)	.95
151-200	0.93 (0.72-1.20)	.59	0.90 (0.69-1.17)	.42
>200	0.85 (0.62-1.16)	.31	0.90 (0.66-1.23)	.50
No. of adults in program				
1-5	1 [Reference]	NA	1 [Reference]	NA
6-10	0.98 (0.83-1.16)	.86	1.04 (0.88-1.24)	.65
11-20	1.16 (0.98-1.37)	.08	1.23 (1.03-1.26)	.02^c^
21-25	1.14 (0.93-1.39)	.21	1.18 (0.96-1.45)	.11
26-30	1.22 (0.98-1.51)	.07	1.26 (1.01-1.57)	.04^c^
31-35	1.21 (0.95-1.54)	.12	1.29 (1.01-1.65)	.04^c^
>35	1.16 (0.91-1.48)	.22	1.24 (0.97-1.59)	.09

Abbreviations: aRR, adjusted risk ratio; NA, not applicable.

^a^ Risk mitigation strategies coded based on endorsement at baseline (May 22-June 8, 2020; no = 0, yes = 1).

^b^ Risk mitigation strategies coded based on endorsement at both baseline (May 22-June 8, 2020) and follow-up (May 26-June 23, 2021) (not endorsed at both = 0, endorsed at both = 1).

^c^ Significant at α < .05.

^d^ Center based = 0 and home based = 1.

We next examined programs that reported practicing different risk mitigation strategies at both baseline and follow-up. Continued child masking (endorsed at both baseline and follow-up) was associated with a 14% lower risk of COVID-19 closures (aRR, 0.86; 95% CI, 0.74-1.00; *P* = .04) compared with programs not practicing child masking at both time points, whereas continued 6-ft distancing decreased COVID-19 closures by 7% (aRR, 0.93; 95% CI, 0.87-1.00; *P* = .05) (Table 4). This finding translates into an absolute risk reduction of 6.4 percentage points (95% CI, 0.6-12.1 percentage points; *P* = .03) for programs practicing child masking.

In the multivariable model in which combined child and adult masking was assessed as the exposure variable, the aRR for both adult and child masking compared with neither child nor staff masking at baseline was 0.85 (95% CI, 0.76-0.97; *P* = .01) and for masking at both time points was 0.87 (95% CI, 0.75-1.01; *P* = .06). Masking only by adults or children was not statistically significant at baseline (adult baseline: aRR, 0.97; 95% CI, 0.90-1.04; *P* = .34; child baseline: aRR, 0.77; 95% CI, 0.45-1.33; *P* = .35) or baseline plus follow-up (adult baseline and follow-up: aRR, 0.97; 95% CI, 0.90-1.05; *P* = .43; child baseline and follow-up: aRR. 0.69; 95% CI, 0.40-1.20; *P* = .19) compared with neither child nor staff masking.

## Discussion

This survey study of a large prospective cohort of health care professionals found that early adoption of child masking in May to June 2020 was associated with a 13% reduction in COVID-19–related childcare program closures during the 1-year follow-up. Furthermore, continued endorsement of child masking at both the May to June 2020 and May to June 2021 timepoints was associated with a 14% reduction in COVID-19 childcare closures when controlling for other risk mitigation strategies, such as social distancing, symptom screening, outside drop-off, and temperature monitoring.

The benefits of masking in preventing COVID-19 spread within kindergarten through 12th grade classrooms are well described.^8,9,10,11^ Masks can be worn safely by young children without compromising respiratory function.^16^ In other studies,^17,18^ childhood infection with other respiratory viruses decreased and asthma symptoms were not reported when masks were worn by preschool children along with other risk mitigation strategies. One reason for this may be that those who wear masks display reduced face touching behavior, a known risk mechanism for respiratory viral transmission.^19^ The federally funded Head Start program requires masks for staff and children as part of a broader COVID-19 prevention plan, a strategy endorsed by the Centers for Disease Control and Prevention and the American Academy of Pediatrics.^20,21^ Most childcare professionals who affirmed child masking also reported their program engaged in multiple other risk mitigation behaviors consistent with this comprehensive approach.

Concerns have been raised regarding the potential for social and developmental delays when younger children wear a face mask for prolonged periods because of lack of recognition of emotional cues.^22,23^ Notably, these are point-in-time studies, and how quickly children adapt and recognize other emotional cues, such as body language, is not known. Evidence suggests that school-age children can identify most emotions in masked faces.^22,24^ Two-year-old children recognize spoken words better through an opaque mask compared with a clear face shield, suggesting verbal communication to infants is not harmed by face masks.^25^ We are unaware of published research on the long-term effects, if any, on intermittent masking. For medical care, most children 4 to 10 years of age did not prefer unmasked health care professionals to masked health care professionals and did not fear health care professionals with masks.^26^

Early adopters of masking may represent a group of highly vigilant programs that emphasized COVID-19 prevention. Surprisingly, we did not find an association between adult masking alone and the prevention of COVID-19–related childcare closures. One possible explanation is that programs that did not endorse strict masking policies were less concerned about COVID-19 in general and less likely to close when there were COVID-19 exposures or cases in the program.

The percentage of programs reporting child masking increased to 33% during the follow-up survey compared with 9% at baseline. However, we did not ascertain specifically when in the study period masking was initiated and whether it was because of a COVID-19 case or as a preemptive measure to prevent closure. Therefore, we did not examine an association between COVID-19 related closures and child masking only in the follow-up survey.

### Strengths and Limitations

This study has several strengths. The main strengths is the prospective data collection from a large national cohort of childcare professionals, which increases the generalizability of our findings. The retention rate at 1 year was high. In addition, the collected data reflected self-reported practices in childcare settings rather than policies that may or may not be adhered to.

The study also has some limitations, including potential respondent bias because childcare professionals were asked about behaviors that were not independently confirmed. Similarly, programs that report mitigation practices at both baseline and follow-up may not have been continuously adhering to these practices, resulting in a biased estimate. We did not ask specifically about childcare program policies regarding masking or criteria for closure. Thus, we do not know what percentage of respondents were adhering to employer guidelines. Different programs may have varied criteria for closure (eg, any COVID-19 exposure in the program vs documented within-program transmission). Our data cannot differentiate between closures that were due to within-center transmission and closures due to imported COVID-19 infection. Both adult and child behavior outside childcare, such as play dates and other social gatherings where child or adult masking are not enforced, also influence COVID-19 cases in congregate settings and therefore the probability of program closure.^27^ Alternatively, adults and children who masked may have engaged in other preventive measures that were not controlled for, such as avoiding travel, reducing the likelihood of importing COVID-19 cases into the childcare program.

A previous study^2^ documenting low SARS-CoV-2 transmission in childcare programs was conducted before the emergence of the Delta variant, which can spread rapidly in elementary school children.^5^ The Delta variant was not the predominant strain circulating in the US during this study period, emerging later. Therefore, our results may underappreciate the value of masking because the SARS-CoV-2 strains circulating during the study period were likely less contagious than the Delta variant.

## Conclusions

Despite these limitations, this large survey study of childcare professionals suggests that masking children 2 years and older can be an important component of risk mitigation strategies for younger children in congregate settings when vaccination is not widely available. Open childcare programs promote in-person early education, beneficial social interactions among children and staff, and financial stability by allowing parents to return to work without interruptions from children in quarantine. Our findings support current national recommendations endorsed by many local and state governments for masking children 2 years and older in childcare programs when community COVID-19 transmission levels are elevated.

## Notes

Supplement. **eAppendix.** Study Survey
