# Supplementary material for: Association of Child Masking With COVID-19–Related Closures in US Childcare Programs
Source: JAMA Netw Open. 2022 Jan 27;5(1):e2141227. doi: 10.1001/jamanetworkopen.2021.41227 (PMC8796014; doi:10.1001/jamanetworkopen.2021.41227)
Supplement: Supplement. — eAppendix. Study Survey [file jamanetwopen-e2141227-s001.pdf]

# Supplementary Online Content

Murray TS, Malik AA, Shafiq M, et al. Association of child masking with COVID-19–related closures in US childcare programs. *JAMA Netw Open*. 2022;5(1):e2141227. doi:10.1001/jamanetworkopen.2021.41227

**eAppendix.** Study Survey

**This supplementary material has been provided by the authors to give readers additional information about their work.**

© 2022 Murray TS et al. *JAMA Network Open.*

**eAppendix.** Study Survey

**SURVEY QUESTIONS**
Below are the questions used in the present analyses as presented in the baseline (May/June 2020) and follow-up (May/June 2021) surveys.

**BASELINE SURVEY QUESTIONS**

1. Do you typically work in child care EITHER as a child care provider who works directly with children OR as a child care director or administrator who works in a program that provides child care? (Please select "Yes" if you work in home-based or family-based child care, nanny/in-home child care, or center-based child care, such as a child care program, Head Start or any other program that provides care for young children.)
   [] Yes
   [] No, I don't provide child care or work in a child care program

2. Which of the following most accurately describes you? *(please check all that apply)*
   [] Female
   [] Male
   [] Non-binary
   [] Transgender
   [] Intersex
   [] I will specify…______
   [] I prefer not to answer

3. Are you of Hispanic, Latino or Spanish origin? *(please check one)*
   [] Yes
   [] No
   [] I prefer not to answer

4. How would you describe yourself? *(please check all that apply)*
   [] American Indian or Alaskan Native
   [] Asian
   [] Black or African American
   [] Native Hawaiian or Other Pacific Islander
   [] White
   [] Other_______
   [] I prefer not to answer

5. How old are you in years?
   [] under 18
   [] 18-24
   [] 25-34
   [] 35-44
   [] 45-54

© 2022 Murray TS et al. *JAMA Network Open.*

[] 55-64
[] 65-74
[] 75-84
[] 85 or older
[] I prefer not to answer

6. Which of the following best describes your child care program or service?
   - [] Center-based Child Care (such as a child care program, Head Start, etc.)
     - [] For-profit center
     - [] Not-for-profit agency center
     - [] School-based
     - [] Head Start or Early Head Start
     - [] Drop-in child care (the children change every day)
     - [] Faith-based child care program
     - [] Other center-based ________
   - [] Home-based or Family Child Care (providing child care in <u>your own home</u>)
   - [] Nanny or In-Home Child Care (providing child care in the <u>children's homes</u>)

7. Did your program <u>ever</u> close for any amount of time because someone (either staff, a child, or someone else) had COVID-19 <u>or</u> had symptoms similar to COVID-19?
   - [] Yes
   - [] No

8. Currently, about how many children are served in your <u>entire child care center</u>? (Please include all children in the entire program site, even children not specifically in your classroom. If you do not know the exact number, please provide your best estimate.)
   a. Total children served in entire child care center?
      - [] 1-25
      - [] 26-50
      - [] 51-100
      - [] 101-150
      - [] 151-200
      - [] 201 or more
   b. Of the number above, how many children are 0 years old?
      - [] 1-10
      - [] 11-25
      - [] 26-50
      - [] 51-75
      - [] 76-100
      - [] 101 or more
   c. Of the number above, how many children are 1-2 years old? [*Same options as (b)*]
   d. Of the number above, how many children are 3-5 years old? [*Same options as (b)*]

© 2022 Murray TS et al. *JAMA Network Open.*

e. Of the number above, how many children are 6 years old or older? [*Same as (b)*]

9. Currently, about how many adults work on a regular basis in your entire child care center? *(Please include all adults in the entire program site, even adults not specifically in your classroom. If you do not know the exact number, please provide your best estimate.)*
   - [] 1-5
   - [] 6-10
   - [] 11-20
   - [] 21-25
   - [] 26-30
   - [] 31-35
   - [] 36 or more

10. Please answer the following questions based on what you or someone else in your classroom or program were able to do throughout most of the month of April, for the days your program was open.

| | | Never | Some days | Most days | Every Day |
|---|---|---|---|---|---|
| a. | Someone screened all children for COVID-19 symptoms at least 1 time per day | [] | [] | [] | [] |
| b. | Someone screened all staff for COVID-19 symptoms at least 1 time per day | [] | [] | [] | [] |
| c. | Someone checked all children's temperatures at least 1 time per day | [] | [] | [] | [] |
| d. | Someone checked all staff temperatures at least 1 time per day | [] | [] | [] | [] |
| e. | All other adults wore a mask or facial covering | [] | [] | [] | [] |
| f. | All children (2 years and older) wore a mask or facial covering | [] | [] | [] | [] |
| g. | Someone ensured that all seating and bedding/cots were at least 6 feet apart at all times | [] | [] | [] | [] |
| h. | The program staggered arrival and pick up times to limit physical congestion | [] | [] | [] | [] |
| i. | All children were picked up and dropped off outside of the program (families did not come inside) | [] | [] | [] | [] |

**FOLLOW-UP SURVEY QUESTIONS**

1. We are sending this survey to you because you completed a survey for us last Spring 2020 and said you were interested in participating in follow-up surveys.

© 2022 Murray TS et al. *JAMA Network Open.*

This survey is for anyone who is either currently employed as a child care provider OR has worked in child care at any point during or just prior to the pandemic. This includes but is not limited to:

- Center-based Child Care Providers
- Home-based or Family Child Care Providers
- Nanny or In-Home Child Care Providers
- Child Care Providers that are OR have been working in-person
- Child Care Providers that are OR have been working remotely or online
- Child Care Providers that have been laid off
- Child Care Providers whose program is now temporarily OR permanently closed
- Other Child Care Support Professionals, such as nurses, therapists, psychologists, consultants, supervisors, etc.

Do you consent to participation in this survey? *(please select one.)* [presented with consent form]

[] Yes, let's go!
[] No, I'm positive I did not complete a child care survey from Yale in Spring 2020 or I've never worked in a child care program.
[] No, I completed the first survey, but I don't want to complete this one.

2. Did either your current or former child care program <u>ever</u> close for any amount of time because someone (either staff, a child, or someone else) had COVID-19 <u>or</u> had symptoms similar to those of COVID-19?
   [] Yes
   [] No

3. Please answer the following questions based on what you or someone else in your classroom or program are currently able to do, for the days your program is open.

| | | Never | Some days | Every Day |
|---|---|---|---|---|
| a. | Someone screens <u>all</u> <u>children</u> for COVID-19 symptoms <u>at least 1 time per day</u> | [] | [] | [] |
| b. | Someone screens <u>all</u> <u>staff</u> for COVID-19 symptoms <u>at least 1 time per day</u> | [] | [] | [] |
| c. | Someone checks <u>all</u> children's temperatures <u>at least 1 time per day</u> | [] | [] | [] |
| d. | Someone checks <u>all</u> staff temperatures <u>at least 1 time per day</u> | [] | [] | [] |
| e. | <u>All</u> other adults wear a mask or facial covering | [] | [] | [] |
| f. | <u>All</u> children (2 years and older) wear a mask or facial covering | [] | [] | [] |
| g. | Someone ensures that <u>all</u> seating and bedding/cots are at least 6 feet apart at <u>all</u> times | [] | [] | [] |

© 2022 Murray TS et al. *JAMA Network Open.*

| | | | | |
|---|---|---|---|---|
| h. | The program staggers arrival and pick up times to limit physical congestion | [] | [] | [] |
| i. | All children are picked up and dropped off outside of the program (families did not come inside) | [] | [] | [] |

© 2022 Murray TS et al. *JAMA Network Open.*
